# Deducing Mucosal Pharmacokinetics and Pharmacodynamics of the Anti-HIV Molecule Tenofovir from Measurements in Blood

**DOI:** 10.1038/s41598-018-36004-z

**Published:** 2019-01-14

**Authors:** Sachin Govil, David F. Katz

**Affiliations:** 1 Department of Biomedical Engineering, Duke University, Durham, North Carolina USA; 2 Department of Obstetrics and Gynecology, Duke University, Durham, North Carolina USA

## Abstract

Microbicide pharmacokinetic (PK) studies typically sample drug in luminal fluid, mucosal tissue, and blood. Blood measurements can be conducted most frequently, serially within subjects. Antiretroviral drugs, however, act against HIV in mucosal tissue/cells. We computationally modeled the extent measurements in blood can predict concentrations in tissue, focusing on the antiretroviral drug tenofovir delivered by a vaginal gel. Deterministic PK models input host and product factors and output spatiotemporal drug concentrations in luminal fluid, epithelium, stroma/host cells, and blood. Pharmacodynamic (PD) analysis referenced stroma/host cell concentrations to prophylactic values; summary metrics were time from product insertion to protection (t_lag_) and degree of protection (PP_max_). Results incorporated host factors characteristic of population variability. Neural nets (NN) linked simulated blood PK metrics (C_max_, t_max_, AUC, C_24_) to mucosal PK/PD metrics. The NNs delivered high-performance mapping of these multiparametric relationships. Given multi-log variability typical of biopsy data for tenofovir and other topical microbicides, results suggest downstream but higher fidelity measurements in blood could help improve determination of PK and create inferences about PD. Analysis here is for a tenofovir gel, but this approach offers promise for application to other microbicide modalities and to topical drug delivery to vaginal mucosa more generally.

## Introduction

Development and delivery of anti-HIV drugs are essential in prevention as well as therapeutic strategies against HIV/AIDS. Several drug delivery systems are currently in development for prevention, employing oral, intravenous, subcutaneous, and topical routes of administration^1–4^. Epidemiologically, oral PrEP (pre-exposure prophylaxis) has achieved a key role in overall prevention strategies^5–7^. However, topically delivered and acting microbicides remain an important prophylactic modality in preventing sexually transmitted HIV, due to the relative ease with which they can be formulated, distributed, stored, and applied for sustained release and “on-demand” use^8–10^. These microbicide molecules may act within the mucosal tissue at the stromal host cells which HIV infects (e.g. anti-retroviral drugs, ARVs) or within the lumen directly against HIV virions (e.g. viral entry inhibitors). Microbicides could have particular impact in resource-limited settings where it is advantageous to develop low-cost, single-use products that can be self-administered and have long shelf lives^11–13^. Microbicides were originally developed for vaginal applications but have since been extended to rectal applications to prevent infection due to receptive anal intercourse^14–16^. Various topical dosage forms have been proposed and are in development, including gels, intravaginal rings, fast-dissolving tablets, films, suppositories, and enemas^17–21^.

Rational design of a topical microbicide product requires consideration and integration of many factors that govern the pharmacokinetics (PK) of its active pharmaceutical ingredient (API). These include drug properties, delivery vehicle properties, dosing regimens, and the anatomical, physiological, and histological characteristics of the vaginal or rectal canals and their underlying mucosal tissues^22,23^. An effective microbicide product is one that establishes sufficient concentration distributions of its API, in space and time, to depress the probability of infection by sexually transmitted HIV (and, potentially, other pathogens) in target tissues^2,3^.

Traditional computational PK modeling of microbicide products has employed empirical approaches in which each compartment is homogenous and drug transport is simplified to a first order exchange and loss between compartments^24,25^. While these approaches help inform our understanding of whole-body microbicide PK, they do not fully capture the physicochemical mechanisms that drive drug mass transport. Those mechanisms include convection and diffusion processes, which depend on the rates of spreading and the diffusion and partition coefficients across different compartments^26^. By using deterministic computational compartmental PK modeling, we can better understand drug delivery pharmacokinetics, and how it is governed by the many factors – physiological, anatomical, histological, pharmacological, behavioral, etc. – inherent in it^26^. The models can objectively account for the multivariate, interacting, non-linear effects which these factors have on drug concentration distributions in space and time. Consequently, they can be used to help improve product design and performance evaluation^27–30^.

This deterministic approach to modeling microbicide products can be extended beyond PK to the pharmacodynamics (PD) of microbicide-host cell interactions^26^. It can be used to translate details of microbicide PK to measures of prophylactic efficacy against infection by HIV. Such translation enables, for example, prediction of the time delay between microbicide product application and subsequent mucosal protection, the degree of such protection, and its duration. Understanding the connection between microbicide product PK and PD can not only help inform rational design of products, but can also help inform users about the optimal timing of product application in relation to coital activity and potential exposure to HIV.

*In vivo* PK studies for microbicide products typically involve sampling of drug in three different compartments: fluid in the lumen (via luminal lavage or Weck-Cel sponges), mucosal tissue (via biopsy), and blood plasma (via venipuncture). Variabilities in concentration measurements in these compartments differ significantly and are much greater in luminal fluid and tissue than in blood. Further, sampling at sequential times within subjects is limited, and may not be possible for luminal fluid and tissue. This limitation is not nearly so severe when sampling blood, which can be performed repeatedly within subjects. While the data from blood are of higher fidelity, it is drug concentrations (for ARV molecules) within the mucosal tissue that are the biologically causative factor in preventing sexually transmitted HIV. Consequently, it is meaningful to question the extent to which measurements in blood can be used to deduce information about concentration distributions of microbicide drug in the infectible tissue.

Historically, this has been addressed indirectly and empirically by regarding blood concentrations as biomarkers for concentrations in tissue, without understanding deterministically the connection between the two. Such an approach has fundamental limitations, however, because the kinetics of drug distributions in blood and tissue are different.

We have conducted a series of analyses of drug delivery to the vaginal mucosa using deterministic computational compartmental mass transport models^23,26,31,32^. Using this modeling framework, we have begun here to address the need to deduce information about key mucosal drug concentrations from data in blood. In so doing, we did not simply explore relationships between concentrations in blood and corresponding concentrations in luminal fluid and tissue, but focused specifically on their relationships to microbicide drug concentrations in stromal host cells – the cells to which drug prophylactic potency data may directly link (e.g. for microbicide molecules that act as reverse transcriptase or integrase inhibitors^3^). Further, we have interpreted the concentrations in stroma with respect to a new prophylactic efficacy measure, termed the percent protected (PP)^23,26^. In doing so, we provided a mapping between the PK in blood to the PK and the PD of protection. Our goal in the analysis here was to obtain initial proof of principle of the possibility of employing a computational tool to translate PK measurements in blood to knowledge of corresponding concentrations in target mucosal tissue.

Our analysis here is for the non-nucleotide reverse transcriptase inhibitor tenofovir (TFV). This drug has a long history of evaluation and application against HIV, initially for therapy of infected individuals (e.g. in the oral product Truvada^33^) and later for application prophylactically against sexually transmitted infections (e.g. in oral pills, topical gels, and intravaginal rings^3,23,34^). Tenofovir is phosphorylated to tenofovir diphosphate (TFV-DP) upon entering cells in the epithelium and stroma. It is primarily the cells in the stroma that have the receptors for HIV that enable the virus to enter and infect them^35^. There are considerable data on the activity, pharmacokinetics, and pharmacodynamics of tenofovir^36^. These have been a valuable reference for our prior modeling work and motivate our focus here. Our goal was to obtain initial proof of principle of the possibility of employing computational tools to translate PK measurements of tenofovir in blood to knowledge of corresponding concentrations of tenofovir and tenofovir diphosphate in target mucosal tissue. We addressed the goal here for a vaginally introduced drug formulation, the tenofovir gel^34^.

In order to elaborate linkages in microbicide concentrations across compartments, a computational tool must be chosen. Multivariate correlation analyses of this general type can be performed in many ways^37,38^. A contemporary alternative to those historical approaches is use of machine learning methodology for non-linear mapping and consequent regression. We have chosen the latter approach, employing feedforward neural nets^39–41^. We report here on our first application of the neural net methodology, implemented here for a vaginal gel delivering tenofovir^34,42^. The analysis works to bridge the gap between what can accurately be measured in blood and the need to translate it to pharmacokinetically and pharmacodynamically salient information about infectible tissue.

## Methods

There are three principle elements of the analysis undertaken: (1) computational compartmental PK modeling of drug delivery to the mucosal tissue and bloodstream; (2) interpretation of drug concentrations in stromal host cells in terms of measures of prophylaxis; and (3) bridging the gap between PK information in blood and PK and PD information in tissue. The modeling incorporates the geometry of the vaginal canal and presence of ambient vaginal fluid, mucosal histological structure, infectible host cell locations and concentrations, and the characteristics of gel coating and the tenofovir molecule itself^26,31,43^. The inferences about PD reference information about drug potency, here the EC_50_ value of tenofovir diphosphate. The PK-PK/PD intercompartment connection is made using feedforward neural nets.

### Drug delivery model to vaginal mucosa - pharmacokinetics

**Figure 1:**
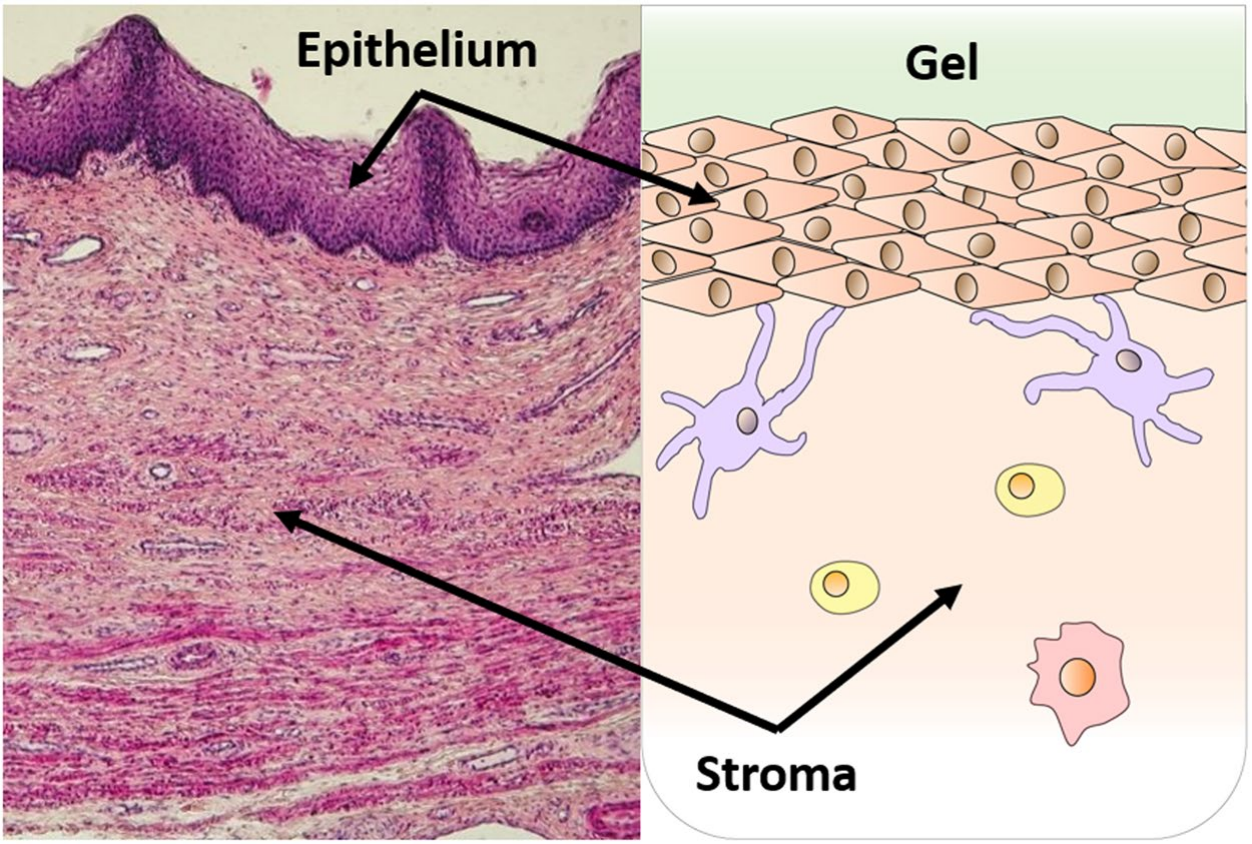
Structure of vaginal mucosa. Histology of the vaginal mucosa depicting epithelial and stromal layers^58^ (left). Schematic of model geometry depicting the gel layer above the mucosal surface (right).

The human vaginal canal has a relatively flat cross-section, in which its width and length are much larger than its undistended height. Its epithelium has a stratified squamous structure that varies in thickness along the canal and also in relation to the phase of the menstrual cycle^44^. The innermost canal opens to the fornix, into which the ectocervix protrudes^45^. Vaginal gels can spread and coat both the body of the canal and its extension to the fornix^46^. Details depend upon several factors, including gel properties, volume, anatomy, and movements of the user^23,26^. The volume of fluid in the vagina also varies^47^. In the analysis here, we have introduced variability parameters characterizing some, but not all, of these factors (epithelial thickness, gel dilution due to ambient vaginal fluid, TFV clearance in the stroma; see below). The geometric model here is rectilinear, with top-to bottom symmetry in luminal fluid contents about the centerline of the canal. We assumed uniform, complete gel coating of the epithelial surface and one-dimensional diffusion of the drug from the gel layer into the tissue^31^. Figure 1 depicts the histology of the vaginal mucosa and an associated schematic of the geometry of the model including the epithelial and stromal mucosal layers.

The core of the computational framework is a system of coupled unsteady diffusion equations that express conservation of mass for drug in the gel, epithelium, and stromal compartments. These equations are coupled to an equation for blood, which is modeled as a spatially homogeneous compartment. These governing equations, and their boundary and initial conditions, are not shown here but were developed in our previous work^31^. Predictions from the model were in good agreement with experimental PK data for humans^31^. The system of equations was solved using MATLAB software^48^ via a finite difference spatial discretization schema.

In order to capture physiological variation between subjects, key anatomical and physiological parameters in the computational model were varied. These included the epithelial thickness (*h*_*e*_), dilution rate constant in the gel for vaginal fluid (*k*_*D*_), and the drug clearance rate constant from the stroma to the blood (*k*_*B*_). The thickness of the stratified squamous epithelium has significant impact on how long it takes drug to reach the stromal compartment (which contains the infectible CD4-positive cells)^44^. The dilution rate takes into account the volume and mixing of ambient vaginal fluid with the gel, as well as gel leakage. There are no published data that measure *k*_*D*_. We have estimated it previously based upon the production rate of ambient vaginal fluid^32,47^ and vary it here based upon the known varying rates of vaginal fluid production^26^. Lastly, the drug clearance rate from the stroma to the blood accounts for loss of drug to the vasculature and lymphatics, assuming a uniform distribution of the capillaries and lymph vessels throughout the stroma. There are, again, no published data based on measurements of *k*_*B*_. We did estimate it previously^31^ and vary it here to allow for variations in architecture of the stromal vasculature (e.g. density of blood vessels). Table 1 depicts the standard values of transport parameters used in the model, as well as the key anatomical and physiological parameters that were varied. This canonical variation produced a dataset of 125 parametrically distinct samples, to use in developing the predictive model.

**Table 1:** Standard values of transport parameters and varied values (in bold) of anatomical and physiological parameters in the model.

Vaginal Model Parameters	Symbol	Value
Gel TFV concentration	C_0_	1 × 10^7^ ng/mL
Diffusion coefficient in gel	D_g_	6 × 10^−6^ cm^2^/s
Diffusion coefficient in epithelium	D_e_	7 × 10^−8^ cm^2^/s
Diffusion coefficient in stroma	D_s_	4 × 10^−7^ cm^2^/s
Partition coefficient of gel/epithelium	Φ_ge_	0.75
Partition coefficient of epithelium/stroma	Φ_es_	1
Gel Thickness	h_g_	400 μm
**Epithelial thickness**	**h**_**e**_	**100, 150, 200, 250, 300, 350, 400 μm**
Stromal thickness	h_s_	2800 μm
Width of vaginal canal	W	3.35 cm
Length of vaginal canal	L	13 cm
**Dilution rate in gel**	**k**_**D**_	**0.386, 0.915, 0.686, 0.915, 1.220 hr** ^**−1**^
**Transport rate to blood**	**k**_**B**_	**0.067, 0.089, 0.119, 0.159, 0.212 hr** ^**−1**^
Clearance rate from blood	k_L_	1.41 hr^−1^
Volume of distribution	V_B_	75 L
Volume fraction of cells in epithelium	φ_e_	0.95
Volume fraction of cells in stroma	φ_s_	0.1
Rate of formation of TFV-DP	k_on_	0.693 hr^−1^
Rate of elimination of TFV-DP	k_off_	0.00413 hr^−1^
Equilibrium ratio of TFV-DP to TFV	n	0.1
Prophylactic concentration of TFV-DP	EC_50_	224 ng/mL

The epithelial thickness (h_e_), dilution rate constant in the gel (k_D_), and stromal transport rate constant to the blood (k_B_) were varied. This parametric variation produces 125 distinct cases.

The fundamental outputs of this model are tenofovir and tenofovir diphosphate concentrations in different compartments. The TFV and TFV-DP concentrations are functions of time and location within compartments (except in blood, in which a volume-averaged value is determined). From this, standard pharmacokinetic metrics (viz. C_max_, t_max_, AUC, C_24_) can be computed for each compartment by taking volume averages (which are comparable to mass averages).

### Vaginal mucosa percent protected – pharmacodynamics

The percent protected (PP) is computed by calculating the fraction of the stromal volume within which local microbicide concentrations equal/exceed a designated target prophylactic value^26,49^. A conservative estimate of 224 ng/mL (500 fmol/mg) was used for the EC_50_ of TFV-DP^50^. We emphasize that model feasibility is not dependent upon this value per se and that it can be varied in expanding the analytical approach. Assuming uniform distribution of host cells in the stromal compartment, this definition of PP is proportional to that based on a corresponding target prophylactic concentration in the host cells themselves.

The PP is a measure of drug pharmacodynamics (PD) and is time dependent. It enables translation of details of microbicide PK to prophylactic efficacy against infection by HIV. The time course of this metric can be characterized by several parameters. Because tenofovir diphosphate has a very long half-life in cells (on the order of days^51^), we focus here upon two of these parameters: t_lag_ – the time between product insertion and maximum protection; and PP_max_ – the absolute value of that protection.

### Feedforward neural net

**Figure 2:**
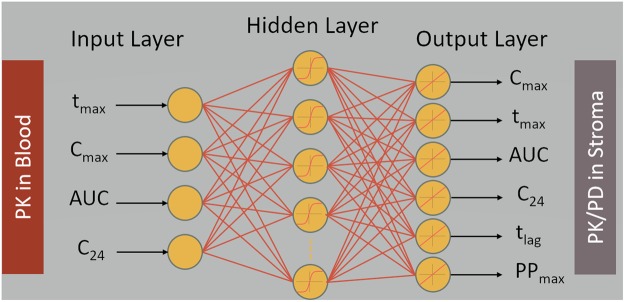
Feedforward neural net architecture. Mapping C_max_, t_max_, AUC, and C_24_ for TFV in blood (PK metrics) to C_max_, t_max_, AUC, and C_24_ for TFV in stroma (PK metrics) and t_lag_ and PP_max_ in stroma (PD metrics) using machine learning.

We used the machine learning technique of neural nets^40^ to deduce concentrations of TFV in the vaginal mucosal stroma from those in blood, in particular, a set of feedforward neural nets. The neural net approach was chosen because of its ability to accurately capture non-linear multiparametric relationships^52^. Further, the feedforward architecture exhibits excellent performance in regression applications^41^. In the net architecture here, the hidden layer was composed of neurons with sigmoidal activation functions, and the output layer was composed of neurons with linear activation functions. A multi-layer perceptron with a single hidden layer containing ten neurons was utilized. The Levenberg-Marquardt algorithm was employed as the training algorithm using the mean-squared error as the optimization criterion^53^. In order to prevent overfitting and to improve neural net generalization, multiple neural nets were created for each output variable (C_max_, t_max_, AUC, C_24_, t_lag_, and PP_max_) and trained starting from different initial weights and biases. These nets were trained on a subset of the computationally generated data, and then tested on a completely independent test set. Those nets for each output variable that generalized best to the independent test set were selected as the mapping functions of choice. Figure 2 is a schematic of this neural net architecture.

## Results

### Pharmacokinetics of drug delivery to vaginal mucosa

**Figure 3:**
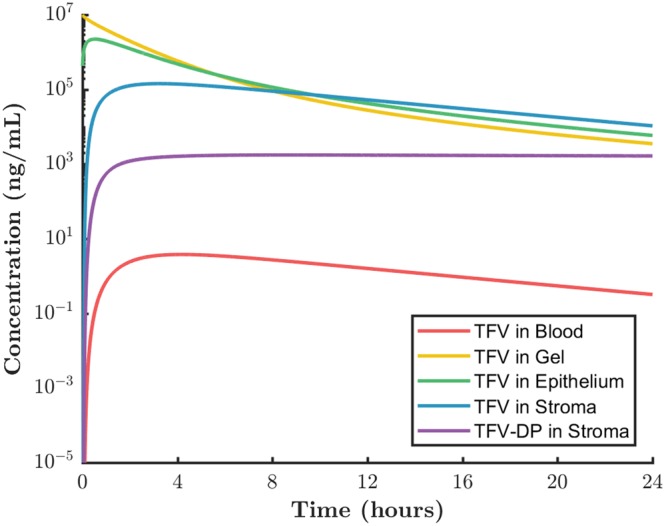
Example PK curves of volume averaged concentrations in different compartments vs. time. Compartments are blood, gel, epithelium, and stroma. Tenofovir (TFV) is shown in all compartments and tenofovir diphosphate (TFV-DP) is shown in the stroma, where it acts against target HIV-infectible cells.

Example PK curves generated from the model are shown in Fig. 3. These were obtained using the standard values of fixed transport parameters and the median values of the varied anatomical and physiological parameters (Table 1).

**Figure 4:**
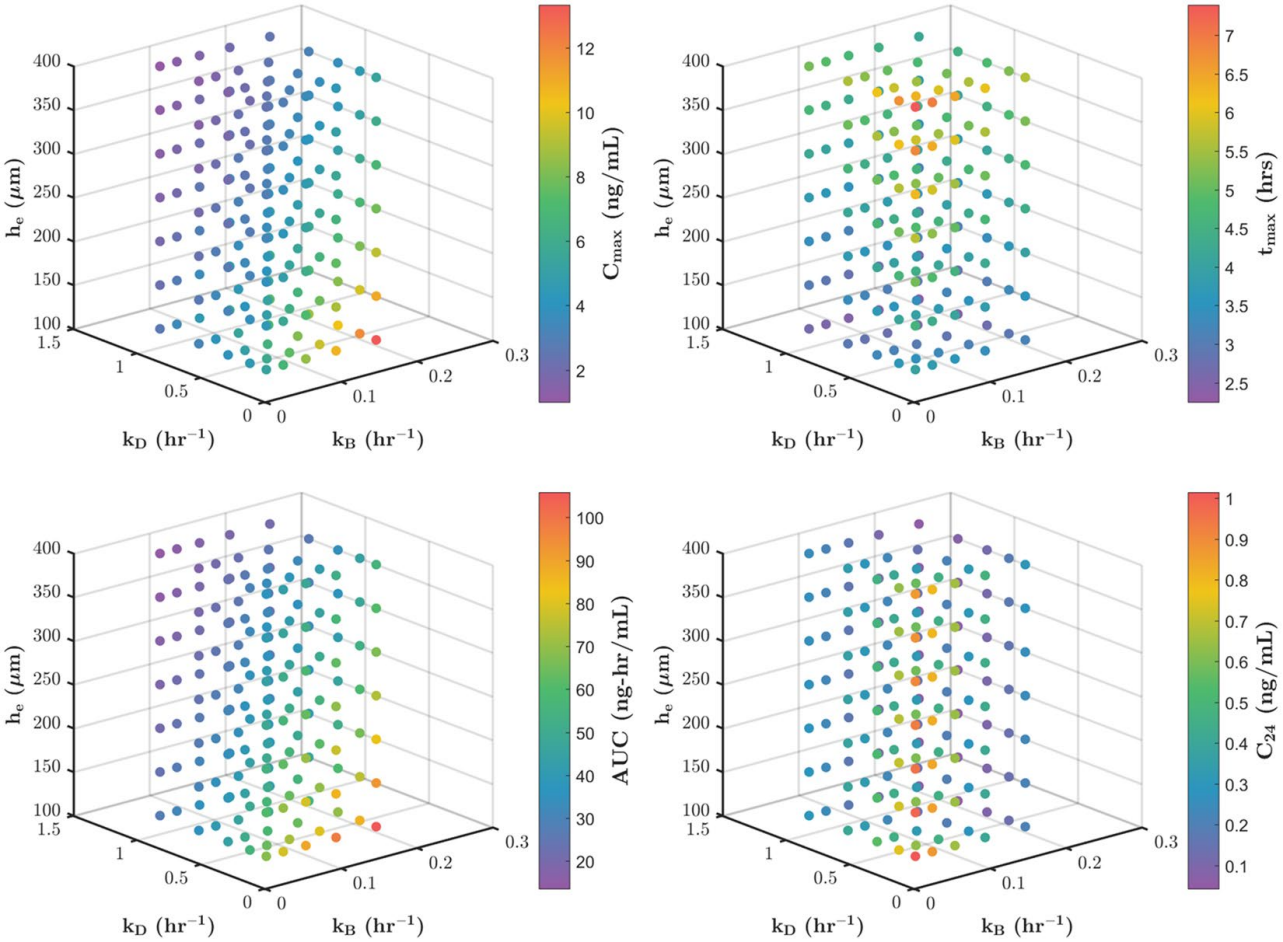
Sample effect of anatomical and physiological parameter variation on TFV PK metrics in blood. The variation involves epithelial thickness (*h*_*E*_), dilution rate in the gel (*k*_*D*_), and drug transport rate from the stroma to the blood (*k*_*B*_).

A set of these PK curves was generated for the blood and stromal compartments, ranging over canonical variations of the parameters governing drug transport, and simulating variability in an experimental PK study. The summary PK metrics C_max_, t_max_, AUC, and C_24_ were calculated for each case. Figure 4 illustrates how the PK metrics in blood were affected by changes in the model parameters – epithelial thickness (*h*_*e*_), dilution rate in the gel (*k*_*D*_), and drug transport rate from the stroma to the blood (*k*_*B*_). Taken together, the plots shown in Fig. 4 show the complex and non-linear dependence of drug concentration distributions on parametric variation.

### Pharmacodynamics of vaginal mucosa percent protected

**Figure 5:**
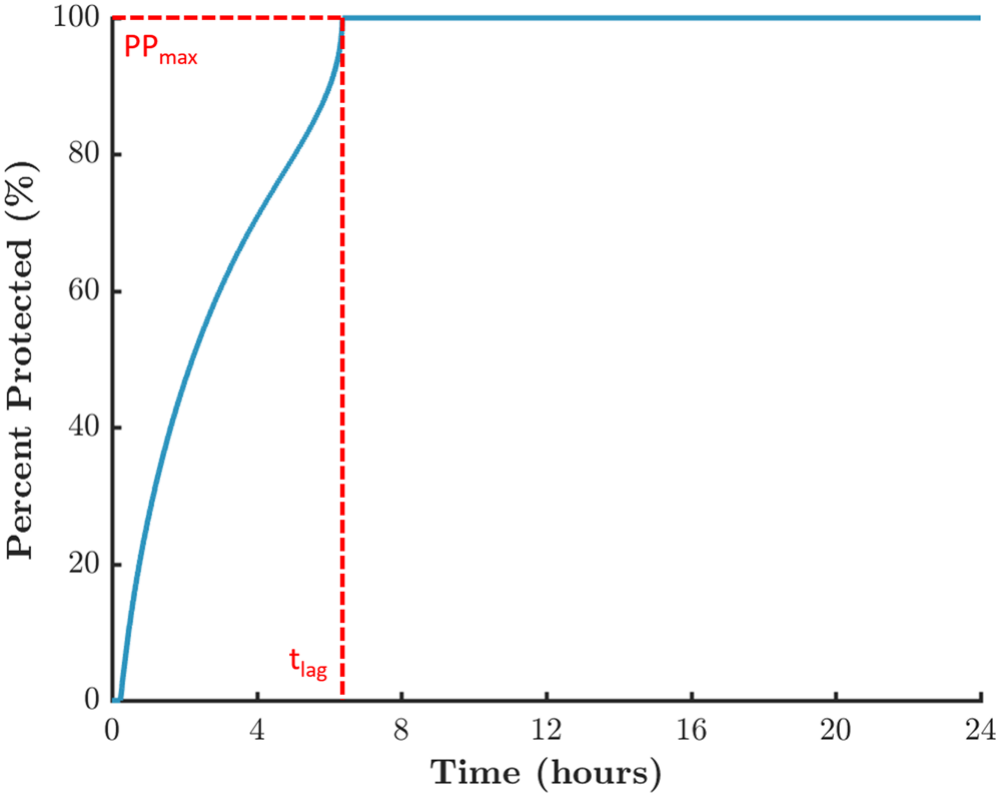
Example curve of the time history of the percent protected (PP) measure of mucosal protection by tenofovir diphosphate against HIV infection. The PP is the instantaneous fraction of stromal volume within which TFV-DP concentration is greater than or equal to a reference EC_50_ value. Here the reference EC_50_ is 224 ng/mL (500 fmol/mg).

Figure 5 is an example curve of the PP for the standard values of transport parameters and the median values of the varied anatomical and physiological parameters (Table 1).

### Neural net regression mapping of PK in blood to PK/PD in stroma

Performance of the neural nets was evaluated using regression of the neural net-predicted values vs. their corresponding PK/PD model-determined values. The set of PK metrics of TFV in blood was used by the neural nets to predict the four PK metrics of TFV in the stroma and the two PD metrics of PP (based on TFV-DP concentrations) in the stroma. The linear fits were uniformly excellent (Table 2).

**Table 2:** Linear fits and performance of neural net mapping.

Metric	Linear Fit	Performance (R^2^)
C_max_	f(x) = x − 40 (ng/mL)	0.99997
t_max_	f(x) = x + 0.0028 (hrs)	0.99998
AUC	f(x) = x − 4.9 × 10^2^ (ng-hr/mL)	0.99998
C_24_	f(x) = x + 6 (ng/mL)	1.00000
t_lag_	f(x) = 0.99× + 0.022 (hrs)	0.98369
PP_max_	f(x) = 0.98× + 1.4 (%)	0.98766

The neural net architecture employed includes six outputs: four PK metrics for TFV in stroma and two PD metrics for PP (based on TFV-DP concentrations) in stroma. In the linear fit, f(x) is the neural net-predicted value for TFV PK and TFV-DP PD metrics in stroma (having input the PK metrics for TFV in blood to the neural net), and x is the corresponding value in the stroma as computed by our computational PK model.

## Discussion

Rational design and performance evaluation of microbicide products are constrained by technical and logistical factors in the sampling of their delivered drug concentrations across multiple compartments. One consequence is that there is substantial variability in the raw PK data and in summary metrics derived from them. The sampling of concentrations in blood is more logistically convenient, may pose less stress to study participants, has fewer technical factors contributing variability in measured concentrations, and can be undertaken serially within experiments much more frequently than sampling in luminal or mucosal compartments. For example, logistical and financial factors sometimes limit sampling in biopsies to two time points after product insertion, typically a relatively short time (e.g. 2 or 4 hrs) and a longer time (e.g. 24 hrs). Deduction of summary PK metrics for mucosal tissue (viz. t_max_, C_max_, AUC, C_24_) from only these two time points is not possible. Even when biopsies are taken more frequently, meaningful deduction of summary metrics can be limited due to high variability. Serial blood sampling over time within an experiment, without disturbing drug concentration in target mucosal tissue or luminal fluid, can provide an adequate number of time points from which to use statistical methods to compute the set of summary PK metrics with better precision than in tissue^54^. Further, it enables repeated measures experimental designs and statistical analyses. This contrasts with sampling luminal lavages or biopsies, which can only be performed once per experiment; thus, repeated measures designs and analyses are not possible, contributing to reduced statistical power per sample size.

The computational simulation here was for a vaginal gel delivering tenofovir. The configuration of the PK model included several simplifications: the geometry of the vaginal canal was rectilinear; the histological structure and fluid content of the canal were uniform along its length; and the gel coating of the mucosal surfaces was uniform along its length. As a result, the dependence of drug delivery with respect to position along the vaginal canal was not evaluated. Concentrations here represent effective longitudinal average values along the canal. In principle, there will be longitudinal variability in drug transport, and our more advanced PK modeling for the tenofovir gel has addressed this^23,26^. Follow up to the analysis here can address those factors. We do note that even for the advanced modeling, the approach here requires an effective longitudinal average of luminal and mucosal drug concentrations, for comparison with the volume-averaged values in blood. There has been experimental analysis of longitudinal dependence of microbicide delivery along the vaginal canal in non-human primates^55^. Statistically significant differences along the canal were not found, but the high variability in experimental PK data compromised the incisiveness of those comparisons.

Results here suggest that tenofovir concentration measurements in blood could be used to deduce salient summary PK/PD metrics for corresponding tenofovir and tenofovir diphosphate concentrations within the vaginal mucosal stroma. By canonically varying parameters in the PK model, we simulated variability in PK data that would occur in a clinical trial of the tenofovir gel (Fig. 4). This provided a database of simulated experimental results with which to develop and test our neural net-based linkage of PK data for tenofovir in blood to PK data for tenofovir in target mucosal tissue. We also linked blood PK metrics to a measure of prophylactic functioning of TFV-DP concentration in tissue, the percent protected (Fig. 5). Our linkage tool was a series of feed forward neural nets, one for each output variable, trained with one set of model simulations and then applied with a different set of simulations (Fig. 2). As seen in Table 2, these neural nets produced excellent linear predictive relationships.

Strictly speaking, there is a time lag in the kinetics of drug concentrations within the mucosal stromal vasculature vs. those at a site of venipuncture. The precise extent of the lag will be drug-specific, depending upon physicochemical properties of the molecule. Our model for the kinetics of drug concentration in the blood compartment accounts for the entire volume of distribution of the blood to help compensate for this lag. Further, blood circulation throughout the body is rapid, tending to reduce the lag, and we suggest that its significance is small as compared to the benefit of more accurate and incisive knowledge of mucosal PK to be gained by the methodology here.

**Figure 6:**
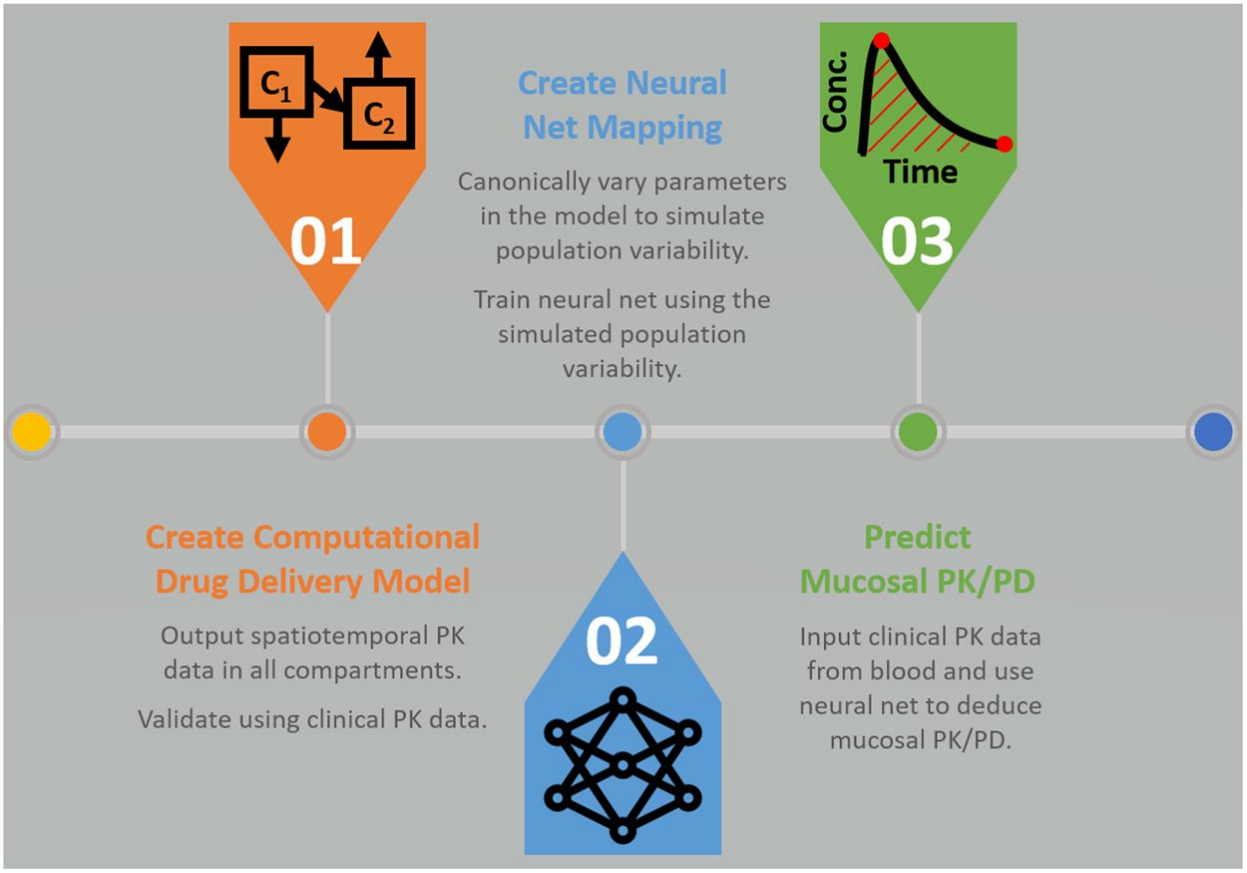
Methodology process diagram. Schematic of steps in implementing neural net to predict mucosal PK metrics and, if data on drug potency are available, deduce resulting mucosal protection against infection (PD).

We emphasize that results here are an initial proof of principle exercise, specifically for the vaginal tenofovir gel. Improvements in the PK and NN modeling might enhance and broaden its potential applicability, but details are likely to have drug and vehicle specific distinctions. It is instructive to consider the applicability of these results in the context of an experimental PK trial setting. This would begin with creation of a deterministic computational compartmental PK model for the test product (e.g. as we have done to date for vaginal gels and films, intravaginal rings, and rectal enemas^31,32,49^). The overarching rationale is that use of this model, together with PK data in blood, would yield improved estimates of mucosal PK metrics for the test product vs. the raw, highly variable mucosal PK data alone. Further, it would do so on a per experimental subject basis. This could improve inferences about central tendencies in the metrics across a study population. Implementation of the methodology would proceed as outlined in Fig. 6.

1. First a deterministic computational PK model would be created (if not already available) for the test product, specific to its properties and those of its active pharmaceutical ingredient. Input parameters to the model would be obtained: (a) by direct measurements – for example, rheological properties governing vaginal deployment of the vehicle^43^ and solubility and transport properties of the drug^56^; and (b) by reference to other studies – for example, clearance rate constants in the circulation from different dosing studies of the same drug. If prior pharmacokinetic data already existed for the product, model predictions would be compared to them, and improvements in the model would be implemented to the extent warranted by the data – for example, refinements of values of parameters such as rate constants.

2. The PK model would then be run while canonically varying salient user-based parameters within it, in a manner akin to that undertaken here. Results would be analyzed to build a neural net predictor of mucosal PK metrics from blood PK metrics, as undertaken here. Goodness of fit of neural net predictions of mucosal PK vs. original model predictions would be evaluated.

3. The neural net would be applied, inputting summary PK metrics for drug concentrations in blood for the test product. This would be performed using data for each participant in the study individually, thereby creating a set of predicted user-specific mucosal PK metrics. From these, central tendencies for population values of the metrics would be computed, a result that is not frequently possible via current temporal sampling of biopsies. Data on drug potency permitting, inferences about PD (as in the percent protected here) would also be deduced.

Results from (3) would provide predictions of mucosal PK across the population of participants in the study, including both central tendencies and variabilities for individual PK metrics. These would be interpreted, together with any direct mucosal PK data, in gauging product performance. The NN approach would subsequently be useful in conjunction with PK data from additional studies. These would be used to further improve the core computational PK model, as in (1) above. Steps (2) and (3) would follow, resulting in enhanced understanding of the mucosal PK for the test product across multiple studies.

We again emphasize that results here are an initial proof of principle exercise. We applied this modeling concept to delivery to the vaginal mucosa of the well-known anti-HIV molecule, tenofovir, delivered by a gel, for which abundant PK data are available, and for which our PK model predictions have agreed well with experimental data in humans^57^. Successful results here, within the confines of the computational framework, are promising. They suggest that this approach might extend to a range of vaginal dosage forms and drugs delivered to the tissues of the lower female reproductive tract.

## Data Availability

The datasets generated during and/or analyzed during the current study are available from the corresponding authors on request.
